# Male cooperation improves their own and kin-group productivity in a group-foraging spider

**DOI:** 10.1038/s41598-022-27282-9

**Published:** 2023-01-07

**Authors:** Bharat Parthasarathy, Marlis Dumke, Marie E. Herberstein, Jutta M. Schneider

**Affiliations:** 1 Institute for Cell and Systems Biology of Animals, Universität Hamburg, 20146 Hamburg, Germany; 2 Department of Biological Sciences, Macquarie University, North Ryde, NSW 2109 Australia

**Keywords:** Experimental evolution, Social evolution, Behavioural ecology, Evolutionary ecology, Ecology, Evolution, Zoology

## Abstract

Cooperation should only evolve if the direct and/or indirect benefits exceed the costs. Hence, cooperators are expected to generate selective benefits for themselves and the kin-group while defectors will impose costs. The subsocial spider, *Australomisidia ergandros*, shows consistent cooperation and defection tactics while foraging. Cooperative individuals are consistently likely to share prey with other group members whereas defector spiders rarely share the prey they acquired. Here, we assess costs and benefits of cooperation, and the causal determinants behind cooperative and defective phenotypes. We constructed experimental kin-colonies of *A. ergandros* composed of pure cooperative or defector foragers and show that pure cooperative groups had higher hunting success as they acquired prey more quickly with greater joint participation than pure defector groups. Importantly, defectors suffered higher mortality than cooperators and lost considerable weight. A social network approach using subadult spiders revealed that foraging tactic is sex dependent with males cooperating more frequently than females. Our results provide a rare empirical demonstration of sex-specific male cooperation that confer individual and kin-group benefits.

## Introduction

Individuals within social groups can benefit from cooperation^1^, however, groups are also vulnerable to exploitation by defectors (cheaters)^2^. Therefore, successful social groups are predicted to have evolved mechanisms that control or offset the costs imposed by defectors^3–5^. Kin selection is one proposed mechanism which minimizes defection within related individuals because cooperation can improve inclusive fitness of the actor^6,7^, and previous studies have validated this claim^8–10^. In parallel to kin selection, game theoretical models predict the ratio of cooperators to defectors to follow a negative frequency dependent selection^11–13^; defectors are likely to reap greater payoffs when the proportion of cooperators is high within the group, and empirical demonstration on microorganisms corroborate these theoretical models^14,15^. Although deciphering the benefits and costs of cooperation and defection is crucial to understand social evolution, it is equally important to unravel the causal determinants, on which selection in cooperation-defection-scenarios ultimately acts on.

The Australian subsocial spider, *Australomisidia ergandros*, is a powerful model to investigate cooperation-defection-scenarios. These spiders live in kin-groups and hunt by ambushing prey. They show two behaviourally consistent foraging types, despite controlling for potentially confounding state-dependent effects such as hunger: (i) A cooperative type, which actively hunts and shares prey with other group members who do not participate in the hunt and (ii) a defector type, which is more likely to feed on the prey captured and shared by cooperators. Defectors are also capable of hunting, but rarely share prey when they do so^16^.

In this study, we unraveled the proximal determinants governing cooperation-defection tactic use in subadult *A. ergandros* colonies. A previous study showed that males in *A. ergandros* contributed significantly in nest construction whereas in the closely related congener, *Australomisidia socialis*, males did not participate in nest construction^17^. Therefore, we hypothesized that sex is a determining factor for cooperative behaviours in *A. ergandros* and that males are more likely to cooperate in prey sharing. Subsequently, we performed additional experiments on juvenile *A. ergandros* colonies to demonstrate the benefits generated by the cooperators on themselves as well as on the kin-group and the costs imposed by defection.

## Results

### Cooperative groups attacked prey more swiftly with greater cooperation

**Figure 1:**
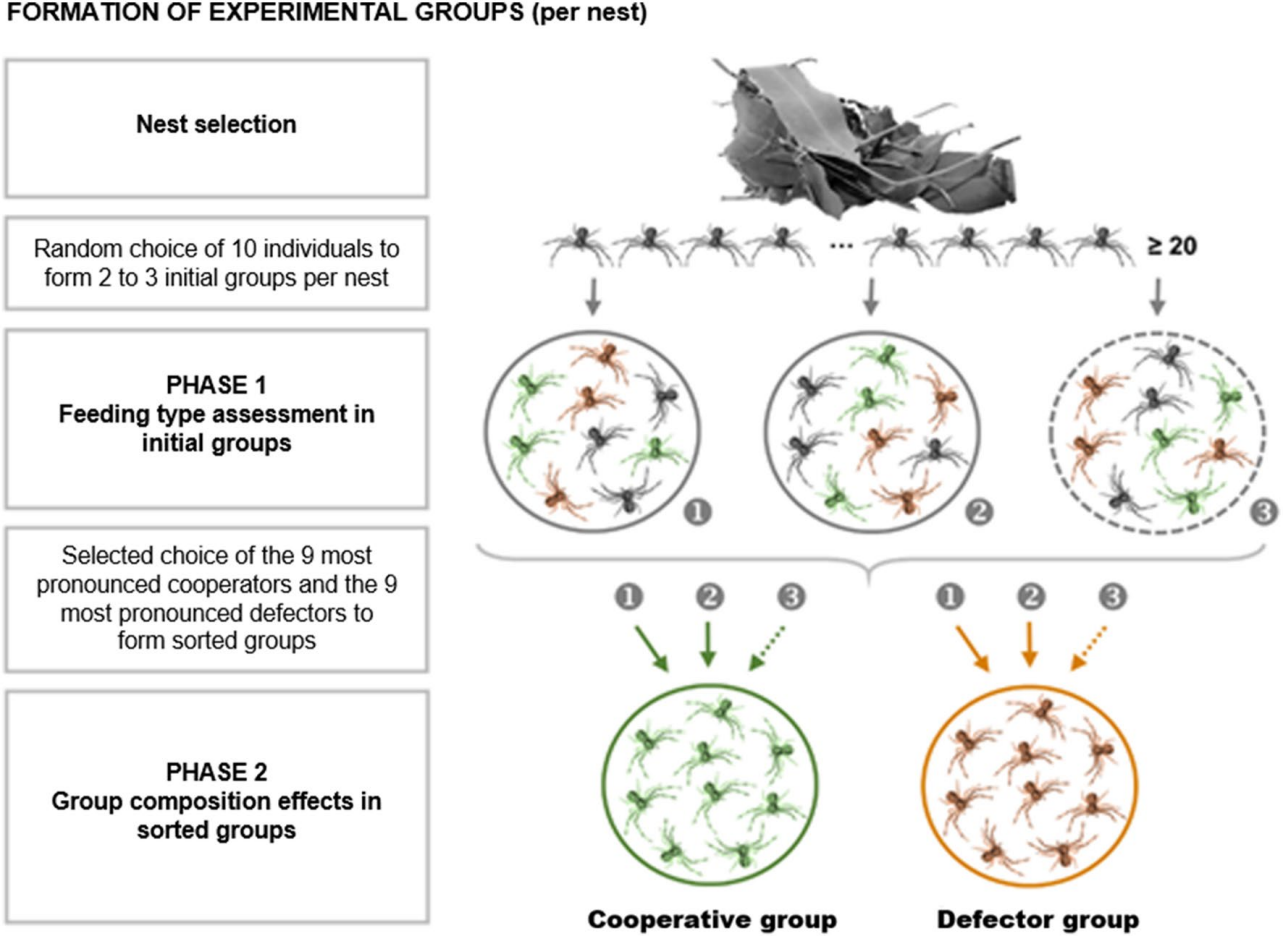
Formation of experimental groups from selected nests (*N*_nests_ = 10) shown for one nest as an example. We formed two to three initial groups per nest for ‘phase 1’, the assessment of individual feeding strategy. We then re-grouped individuals according to their feeding strategy (cooperator or defector, marked green and brown respectively) in the initial groups. Grey spiders did not show pronounced cooperator or defector behaviours. In the sorted groups, we tested for group composition effects on social foraging behaviour and individual fitness payoffs.

**Figure 2:**
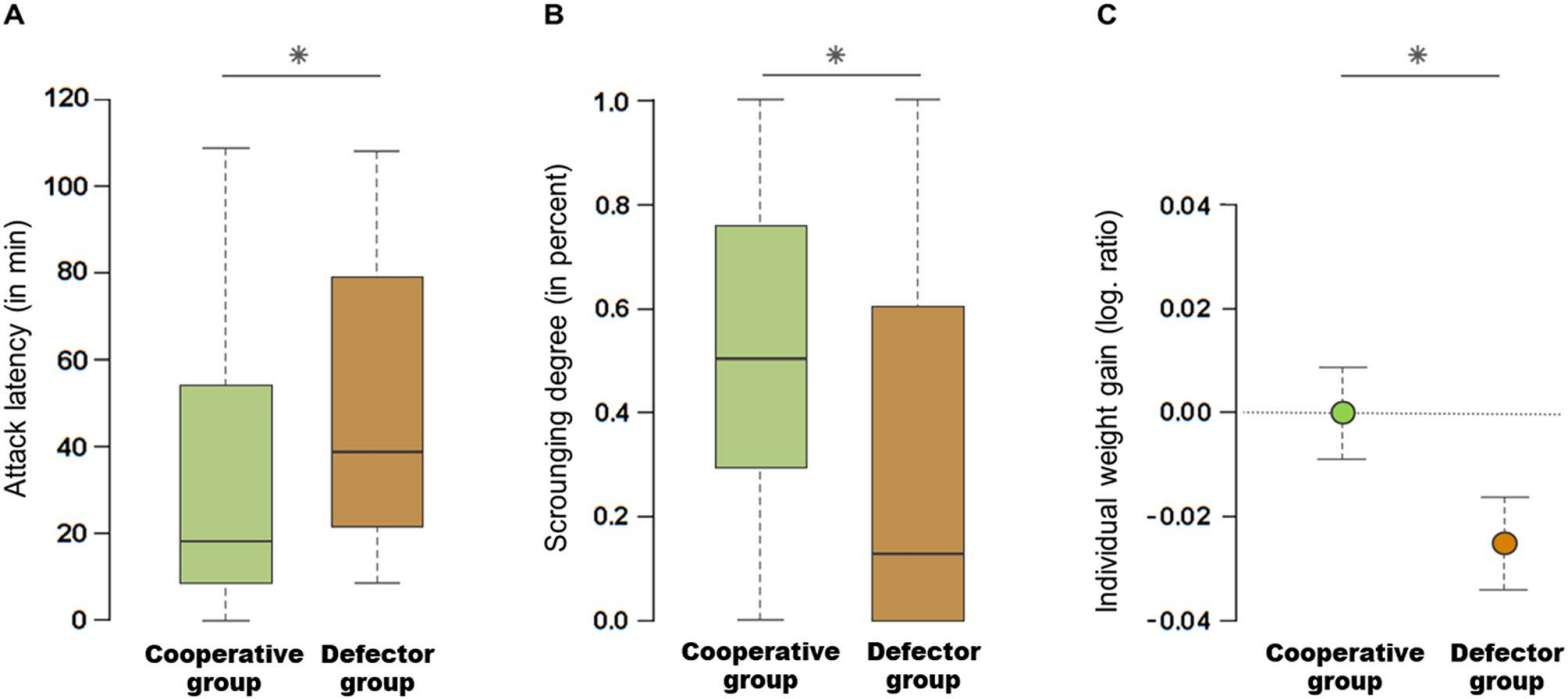
Group composition effects on social foraging behaviour and individual fitness payoffs. **A**, **B**: The effects of group composition (cooperative group ‘c’ or defector group ‘d’) on attack latency (**A**) and the extent of prey sharing (**B**); only successful attacks are considered (c: *N* = 27, d: *N* = 21). The boxplots show median, upper and lower quartiles and interquartile range (1.5 times). **C**: The effect on individual weight gain (c: *N* = 38, d: *N* = 31) presented as mean (circle) ± SE (whiskers). * indicates significant difference between group compositions (see also Table 1).

Our experiments involved assessing the feeding strategy of individuals followed by constructing experimental kin-groups composed of only cooperators or defectors (Fig. 1, methods and supplemental methods sections). Subsequently, we compared four components of attacking behaviour between cooperative groups (c) and defector groups (d): hunting success, latency to attack prey, joint participation (cooperation) in hunting and numbers of spiders sharing prey (scrounging degree). The effect of group composition on hunting success was not significant even though cooperative groups captured more prey than defector groups (Table 1). Over seven feeding trials per group, we recorded 27 successful attacks in cooperative groups and 21 successful attacks in defector groups (mean no. of successful attacks/group ± SE; c: 5.40 ± 0.51, d: 4.20 ± 0.97). Interestingly, group composition had a strong effect on *attack latency* (Fig. 2A, Table 1): prey was captured notably faster in cooperative groups than in defector groups (mean attack latency ± SE; c: 34.04 ± 6.08 min, d: 49.76 ± 7.09 min). Moreover, cooperative groups tended to perform more joint attacks (Table 1). More than one individual performed 70.37% of the 27 successful attacks in cooperative groups, whereas this applied to only 33.33% of the 21 successful attacks in defector groups (mean no. of joint attacks/group ± SE; c: 3.80 ± 0.49, d: 1.40 ± 1.17).

**Table 1:** The effect of group composition on social foraging behaviour and individual fitness payoffs.

Variable	Variable definition	Analysis	Test statistics	*P*-value
**Social foraging behaviour (data per trial and group):**				
*Attack success*	Specified as 1 if prey was successfully attacked, i.e. subdued and eaten (binomial)	GEE (binomial family)	Wald χ^2^ = 0.87	*P* = 0.352
**For successful attacks (*N***_**c**_** = 27, *N***_**d**_** = 21):**				
*Attack latency*	Time in minutes until the first individual attacked (maximum 60 min)	GEE (gamma family)	Wald χ^2^ = 6.77	***P* = 0.0093**
*Joint attacks*	Specified as 1 if two or more individuals participated in the attack (binomial)	GEE (binomial family)	Wald χ^2^ = 2.80	*P* = *0.0941*
*Scrounging degree*	Number of non-attackers that fed with attackers in relation to the total number of non-attackers	GEE (cbind, binomial family)	Wald χ^2^ = 28.20	***P* < 0.0001**
**Individual fitness payoffs (data per individual)**				
*Mortality*	Specified as 1 if the individual died during the experiment (*N*_c_ = 45, *N*_d_ = 45)	Chi-squared test	χ^2^ = 4.14	***P* = 0.0463**
*Weight gain*_*2*_	= *log* (*end weight*_2_ / *start weight*_*2*_), for all living individuals that were weighted excl. two outliers (*N*_c_ = 37, *N*_d_ = 30)	GLS (Gaussian)	*L*-ratio χ^2^_3_ = 4.85	***P* = 0.0277**

Significant *P*-values are indicated in bold, trends in italic. The abbreviations ‘c’ and ‘d’ in the statements of sample sizes mean cooperative groups and defector groups, respectively. The lower-case number ‘2’ in the formula for *weight gain* indicates that individual weight was taken in the 2^nd^ experimental phase (see methods for details).

### Selfish defectors shared prey less frequently with group members

Cooperator groups and defector groups differed significantly in the extent of prey sharing, which we measured per attack as the *scrounging degree* (Table 1, Fig. 2B). Prey sharing was much more pronounced in cooperative groups, where 48.99 ± 5.90% (mean ± SE) of the respective non-attackers fed on a given prey item. This proportion fell to 27.67 ± 8.68% in defector groups. The difference in prey sharing between group compositions was also reflected by absolute values (mean no. of feeding non-attackers ± SE; c: 3.15 ± 0.42, d: 1.62 ± 0.40).

### Cooperation generate individual and group benefits

We measured individual and kin-group benefits generated by cooperation as mortality and individual weight gain of spiders. Mortality was considerably lower in cooperative groups (Table 1): only three cooperative group members (6.68%) died—compared to eleven defector group members (24.44%; mean no. of deaths/group ± SE; *c*: 0.60 ± 0.40, d: 2.20 ± 0.66). Interestingly, there was a significant effect of group composition on the weight gain of individuals (Table 1, Fig. 2C). On average, cooperative group members maintained their weight, while defector group members lost weight (mean individual weight gain (as logarithmic ratio) ± SE; c: 0.000 ± 0.077, d: −0.026 ± 0.080). The absolute per-capita weight change amounted to 0.01 ± 0.07 mg (mean ± SE) for cooperative group members and −0.21 ± 0.07 mg (mean ± SE) for defector group members. These results demonstrate that defectors impose costs to the kin-group.

### Males are more likely to cooperate

**Figure 3:**
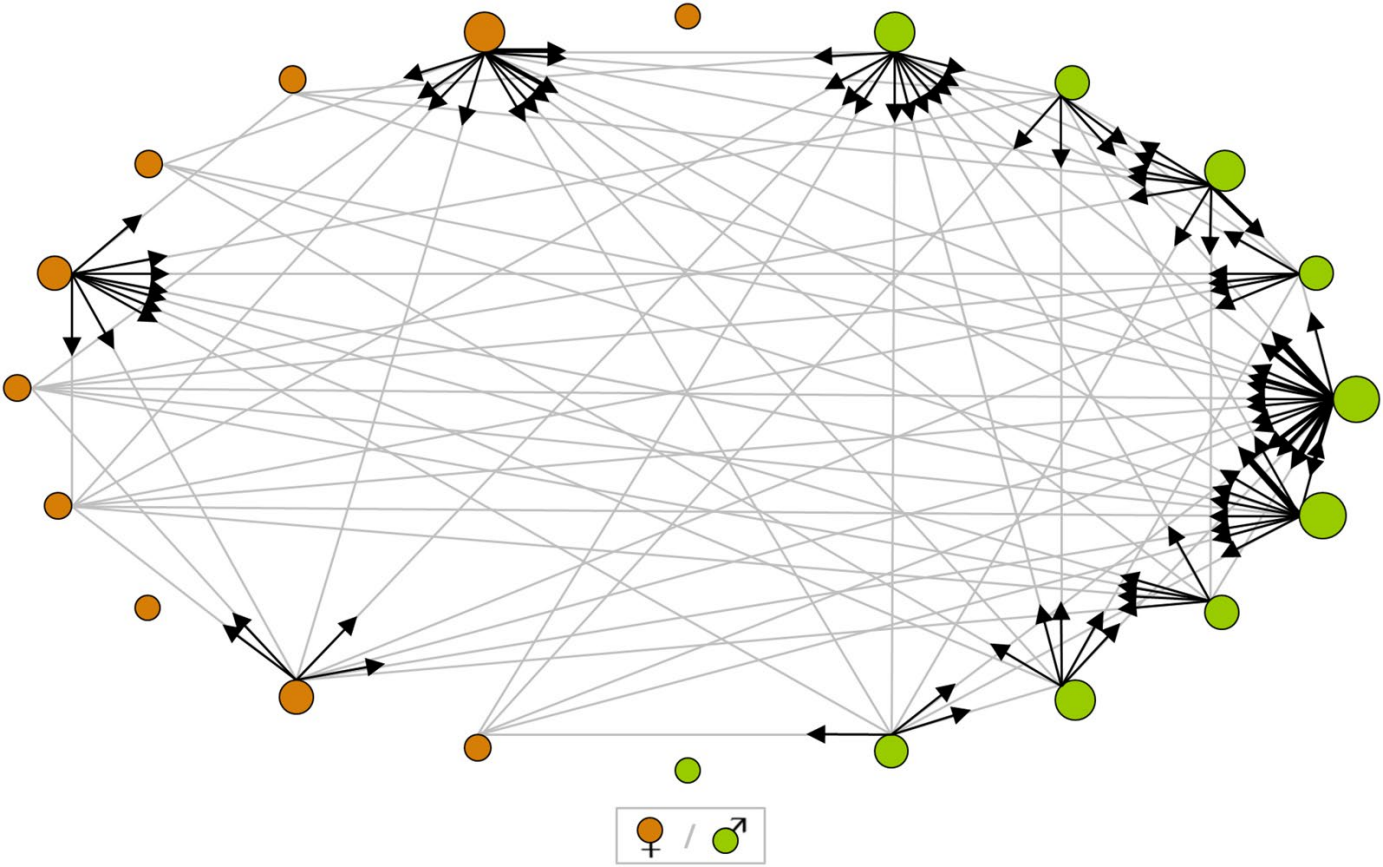
A social network showing the differences in cooperative tendencies between males and females in *A. ergandros*. The graph shows the foraging interactions in one exemplary group of ten males (green nodes) and ten females (brown nodes) recorded over ten repeated feeding trials. A line between two nodes indicates that one individual shared its prey with the other, the respective arrow specifies who acquired food for whom. The number of outgoing arrows per node reflects the food acquisition and sharing tendency of the particular individual in terms of spread over different group members. The node size reflects the frequency with which the individual acquired and shared prey over the duration of the feeding experiments.

To decipher the proximate causes determining cooperation vs. defection, we created experimental groups from 3 subadult colonies consisting of ten males and ten females. We found that males had significantly higher cooperative tendencies than females (Monte Carlo test, *P* = 0.045; Fig. 3). Specifically, the individual *out-strength* (*σ*), a network measure incorporating the frequency and the number of group members an individual produced for, was higher for males (m) than for females (f) in all groups (sex-specific mean ± SE; g1: *σ*_m_ 15.98 ± 4.27 > *σ*_f_ = 10.10 ± 2.94, g2: *σ*_m_ = 9.20 ± 3.23 > *σ*_f_ = 7.80 ± 3.10, g3: *σ*_m_ = 7.70 ± 2.06 > *σ*_f_ = 2.50 ± 1.41). The sum of the within-group differences in sex-specific means amounted to *A* = 12.39. Equal or higher values for this test statistic *A* were achieved in only 450 of 10,000 Monte Carlo randomizations of the data (interval for *A* with 10,000 random. = [−13.86, 14.07], mean random *A* = 0.07). This implies that the detected pattern of cooperation would be unlikely to occur independent of sex.

## Discussion

In group living animals, individuals that cooperate can incur energetic costs of helping conspecifics^12^, but can also confer ancillary benefits to the group. In this study, we unequivocally demonstrated that members of cooperative groups of the Australian subsocial spider, *Australomisidia ergandros,* benefited from improved kin-group productivity. By constructing experimental groups consisting of only cooperators vs. only defectors, we showed that cooperative groups showed greater joint participation in hunting prey more swiftly, suggesting improved hunting success that can benefit the individual and the entire foraging group. We also demonstrate the costs suffered by defectors. (i) Pure defector groups showed approximately four times higher mortality than cooperative groups and (ii) spiders within defector groups lost weight over the experimental duration. Interestingly, we discovered that males were more likely to cooperate by initiating hunts more frequently and sharing food whereas females were more likely to scrounge and rarely shared food. Therefore, we deciphered the benefits and costs of cooperation and defection in *A. ergandros*, and uncovered that sex is a proximal determinant governing the persistence of consistent foraging strategy in this species.

We demonstrated that cooperative behaviour is sex-linked and that males are more likely to cooperate by acquiring and sharing food for the kin-group. This cooperative tendency is more variable among females because few females share and produce food for other members within the group (Fig. 3). Therefore, it is likely that the number of cooperators determine group success. Males sharing food with females are known in a wide range of taxa^18–22^. Prey sharing is often explained as exchange of resources for mating opportunities. However, in *A. ergandros*, the above explanation may not be plausible because: (i) males shared food with other males within the group and (ii) females do not prefer to mate with siblings and are frequently polyandrous^23^. However, genetic data suggest some degree of inbreeding and males may mate within the natal kin-group before they disperse^23^. As the extent of outbreeding is currently unknown, it is difficult to speculate if the even sex ratio in this species, which is at the brink of sociality, is a consequence of selection acting on males for their work or can be sufficiently explained by regular outbreeding^17^. Note that all social spiders are characterized by inbreeding and strong female biased groups^24^.

It is also difficult to explain why most females belonged to the defector type and do not share prey with other group members. One account for this lack of cooperation among sisters could be that only one female can inherit the maternal nest and that sisters compete for this possibility. Females reproduce solitarily and adult females disperse to found new nests. Fast developing and well-fed females may increase their chances of successful reproduction. Females with sufficient food reserves will have more energy for the process of building a new nest and are more likely to survive a period of starvation until the nest is functional. In the social spider *Stegodyphus dumicola*, females differ in body size and in developmental time to reach maturity due to competition for resources between colony members^25–27^. Male *Stegodyphus* appear to exclusively mate inside their natal colony and their reproductive success is likely less condition dependent than that of their sisters. Moreover, in social *Stegodyphus* spiders, allomaternal brood care and shared web-construction may facilitate cooperation among sisters. However, *A. ergandros* neither show allomaternal brood care nor build shared capture webs and thus, intra-sex competition among females might explain this lack of cooperation among sisters. On the contrary, males neither found new nests nor provide brood care and therefore, selection might have favoured those colonies in which males hunted and shared food with their sisters. We speculate that male cooperators might be hunting specialists because our experiments on juvenile colonies showed that cooperator types attacked prey more quickly and participated in more joint attacks, both of which are important requisites for successfully subduing a freely moving prey in the wild.

In conclusion, we showed that cooperation by producing and sharing food has clear advantages in the group living spider, *A. ergandros.* Cooperation-defection-strategies of individuals remained consistent among the pure cooperative and defector groups. We found no evidence that spiders strategically invested in defector-like behaviours in pure cooperator groups and vice-versa over the duration of the experiments. Defector types, which are mostly females, rely on male cooperators that initiate hunts more quickly, show greater cooperation in hunting and likely capture more prey in the wild. After colony foundation, defector females hunt and share prey with their offspring^28^. Thus, defectors are capable of sharing prey with their offspring, but rarely do so with their siblings. Future studies are necessary to interrogate the ultimate reasons for female defection within the kin-group.

## Material and methods

### Study species and spider collection

*Australomisidia ergandros* is a subsocial spider inhabiting South-Eastern Australia. They live in communal kin-groups in nests usually built with leaves from Eucalyptus trees bound by silk threads. Group size usually ranges from 5 to 45 spiderlings. Groups are comprised of the offspring of a single female who provides maternal care until her death^28^. Offspring continue to live in groups for 5 to 7 months after the mother’s death^29,30^. One of the females inherit the natal nest while the remaining females disperse to found new nests. It is not entirely clear if *A. ergandros* inbreed with natal kin or if spiders show a mandatory pre-mating dispersal.

We collected 29 *A. ergandros* nests from a population along Yass River Road in New South Wales, Australia (34° 55′ 20.50′′ S, 149° 6′ 15.53′′ E) in February 2016. At this time of year, the spiderlings are very young and the presence of immigrants, who might influence the extent of social foraging, is improbable^9,31^. For our experiments, we transferred the original nests to the laboratory at Macquarie University in Sydney.

#### Group composition effects

Our experiments spanned a duration of 56 days. To investigate group composition (cooperators vs. defectors) effects, we first assessed the hunting types of individuals within ‘initial’ groups (phase 1) and subsequently composed and tested ‘sorted’ groups of cooperators or defectors only (phase 2). The formation of initial groups was dictated by special requirements. Basically, we randomly selected up to 30 individuals per original nest and split these individuals into two to three initial groups of ten (N_nests_ = 10, N _groups_ = 25). Each selected individual received a unique color mark (©Plaka-Farbe) and was weighed to the nearest 0.01 mg on an electronic balance (Mettler Toledo New Classic MS). Each group was then transferred to a petri dish (100 mm in diameter) which served as the test arena for the hunting type assessment. An acclimatization period of four days ensured that the spiders weaved silk threads which amplify vibrations by prey^32^.

#### Phase 1

We assessed hunting types with a modified version of the ‘communal feeding experiment’ originally used by Dumke et al.^16^ to establish hunting specialization in *A. ergandros*. For each initial group, we completed 7 feeding trials over 24 days (1 trial every 4 days), during which we offered living *Musca domestica* flies and observed the foraging behaviour of all group members (Fig. 1). Each fly was weighed before being placed into the petri dish and either removed after two hours if not captured, or after two hours post capture. For each trial, we documented the attack latency, the attacker IDs and the IDs of the feeding individuals in 10-min intervals over two hours. From these data, we determined the feeding frequency of each individual (i.e. the number of trials it was feeding) and calculated the proportions to which it cooperated vs. defected. We thus obtained comparable quantifications of hunting types^16^. All individuals except those that died during the assessment (56 of 250 spiders) were weighed two days after the last trial to assess weight gain1 (= log (end weight1/start weight1^33^).

#### Phase 2

Following phase 1, we regrouped individuals into ‘sorted’ groups of cooperators or defectors only, and this time gave three days acclimatization time since the re-grouping took one day. We formed experimental cooperator groups by selecting nine to ten individuals with the highest cooperating tendencies from the original colony. Next, we formed experimental defector groups analogously from that same pool (Fig. 1). Thus, we achieved paired relatedness between cooperator groups and defector groups, to control for nest origin and nest experience (matched pairs design). We further ensured comparability of cooperator groups and defector groups in the individuals’ physical state (details in Supplementary Methods). Owing to mortality in three nests and restricted possibilities to realize balanced conditions between groups in two nests, we could establish five cooperator-defector group pairs with nine individuals per group.

To explore group composition effects on social foraging behaviour and individual fitness payoffs, we tested each sorted group over another seven feeding trials over 24 days. The trials were conducted in exactly the same manner as for the feeding type assessment (phase 1). From the recorded data (attack latency, IDs of attackers, IDs of feeding individuals), we calculated a set of variables that quantified social foraging behaviour (data points per trial and group). To examine individual fitness payoffs, we checked the petri dishes for dead individuals and noted their IDs prior to every trial. As an additional fitness payoff measure for those individuals still alive at the end of phase 2, we determined individual weight gain2 (= log (end weight2/start weight2)).

#### The role of sex in cooperator vs. defector types

To examine the role of sex in cooperation-defection scenarios, we collected another eight nests from Yass River Road in June 2016. Around this time, *A. ergandros* individuals reached the subadult stage, at which sex can be visually determined^17^. Three nests contained subadult males and females in sufficient numbers, so that we formed three groups, each with ten males and ten females from the same nest (in total: N _males_ = 30, N _females_ = 30). All group members were weighed and color marked before they were tested in another, extended feeding type assessment over ten trials.

Based on the IDs of attackers and individuals that hunted in these trials, we generated social network graphs and visualized the foraging interactions within groups^31,34,35^. Individuals were represented by ‘nodes’; a directed line (‘edge’) was drawn from one node to another if the specific individual had cooperated by sharing prey with the other. The lines received weights reflecting the frequency of the respective interaction. We quantified individual prey sharing tendencies using the node-level metric out-strength: the weight sum of all outgoing edges from a particular node^35^. This metric comprehensively reflects an individual’s prey sharing tendency, as it incorporates the frequency and the spread of prey sharing behaviour. To visualize social networks and calculate the individuals’ out-strengths, we used the software UCINET 6^36^.

### Statistical analyses

All model analyses were performed in R version 3.2.2, whereas all social network analyses were conducted in UCINET 6^36^.

#### Group composition effects

We modelled the effect of group composition on social foraging behaviour separately for each response variable with binomial or gamma GEEs (generalized estimation equations). GEEs are adequate to analyse data from repeated measurements over time within same groups because they allow adjustment for the dependence of these measurements^37^. Defining the dependence structure of our data, we set sorted-group ID as a grouping variable and specified the temporal correlation AR-1. Group composition constituted the explanatory variable of interest, fly weight and group size were included as additional variables to control for prey mass and mortality. An exception was the model for the scrounging degree, in which group size was controlled by the variable itself. We assessed the significance of group composition effects by dropping each explanatory variable in turn and then comparing the full model to its nested models based on Wald test statistics. The least significant variable was removed, and model comparisons were repeated until all remaining variables were significant.

Mortality was compared between cooperator groups and defector groups using a Chi-squared test. The difference between group compositions in individual weight gain2 was analysed in a GLS (generalized least squares) model that incorporated an exchangeable correlation structure with sorted-group ID as the grouping variable.

#### Sex differences

We conducted a node-based Monte Carlo randomization test to determine whether the observed difference in mean out-strength between sexes deviated significantly from the difference expected if producing associations occurred randomly and hence independent of sex. The observed data were shuffled in 10,000 node-label randomizations that preserved group membership. The sum of the differences between mean male out-strength (σ_m_) and mean female out-strength (σ_f_) within groups was used as the test statistic A $A = \sum\nolimits_{(i = 1)}^{3} {\left( {\sigma_{{(m_{i} )}} - \sigma_{{\left( {f_{i} } \right)}} } \right)}^{ - }$, where i denotes group identity. To produce a probability value, we compared the observed test statistic to the distribution of random test statistics drawn from the 10,000 Monte Carlo simulations^34^.

## Supplementary Information

Supplementary Information.

## Data Availability

The datasets generated and/or analysed during the current study are available in the Mendeley repository (https://doi.org/10.17632/cjpxbn8v4k.1).
