# Supplementary material for: Male cooperation improves their own and kin-group productivity in a group-foraging spider
Source: Sci Rep. 2023 Jan 7;13:366. doi: 10.1038/s41598-022-27282-9 (PMC9825364; doi:10.1038/s41598-022-27282-9)
Supplement: Supplementary file 1 — Supplementary Information. [file 41598_2022_27282_MOESM1_ESM.docx]

**SUPPLEMENTARY METHODS**

**Special requirements regarding group formation**

The experimental procedure that we followed to investigate group composition effects in *Australomisidia ergandros* consisted of two phases: firstly, the assessment of individual feeding types (cooperator or defector) in ‘initial’ groups, and secondly, the assessment of group composition effects in groups composed of cooperators or defectors only (see also Figure 2). To be able to compose and compare these sorted groups from the pool of assessed feeding types, several requirements had to be considered in the formation of initial groups: For comparability, the sorted cooperator-groups and defector-groups had to be of equal sizes. This required the identification of similar numbers of cooperators and defectors in the initial groups. Furthermore, all experimental groups had to fulfill the natural condition of within-group relatedness.

To meet the above requirements, we selected nests with at least 20 individuals of a body size large enough (body length of 2 to 2.5 mm) to be color-marked (*N*_nests_=10). We formed two to three initial groups per nest (Figure 2), such that every group contained nine to ten randomly selected yet related individuals (*N*_groups_=25). We assessed feeding types in this group size, as a previous study demonstrated balanced feeding-type ratios for that group size (details in *16*). To keep all variables but group composition constant between the two experimental phases, we also assessed group composition effects in groups of nine to ten.

**Comparability of cooperator-groups and defector-groups**

We ensured that a cooperator-group and its related defector-group were comparable in terms of the number of individuals chosen from each initial group as well as similar in average individual *weight gain_1_* and average individual weight after the first phase (*end weight_1_*). With this, we aimed to avoid group composition bias in the individuals’ physical state. We statistically tested for the similarity of paired groups in these traits with Wilcoxon signed-rank tests.
